# Precise Discrimination Between Rape Honey and Acacia Honey Based on Sugar and Amino Acid Profiles Combined with Machine Learning

**DOI:** 10.3390/foods15010070

**Published:** 2025-12-25

**Authors:** Chenyu Sun, Fei Pan, Wenli Tian, Zongyan Cui, Xiaofeng Xue, Yitian Xu

**Affiliations:** 1 College of Science, China Agricultural University, Beijing 100083, China; forever_to0325@cau.edu; 2 State Key Laboratory of Resource Insects, Institute of Apicultural Research, Chinese Academy of Agricultural Sciences, Beijing 100093, China; yunitcon@yeah.net (F.P.); leroytian@126.com (W.T.); 3 Technology Center of Qinhuangdao Customs, Qinhuangdao 066004, China; ciqqhd@126.com

**Keywords:** honey variety identification, sugar, amino acid, Multilayer Perceptron (MLP), SHAP interpretability analysis

## Abstract

Honey variety authentication is critical for ensuring market integrity and protecting consumer rights, especially for high-value unifloral honeys, such as acacia honey, which are frequently adulterated with low-value alternatives such as rape honey due to their similar visual appearance. The aim of this study was to develop a method for precise discrimination between rape honey and acacia honey using their chemical profiles combined with machine learning. A total of 542 honey samples were collected from major beekeeping regions in China. Targeted quantification of 12 sugars and 20 amino acids was performed using UPLC-MS/MS. Multivariate analysis revealed significant differences in sugar and amino acid compositions between the two honey types, though partial samples overlapped due to chemical similarity. Six machine learning algorithms, including the Multilayer Perceptron, were employed for classification. Optimization was performed via 10-fold cross-validation and ADASYN oversampling, yielding optimal performance of 98% and 100% prediction accuracies for rape honey and acacia honey, respectively, on the independent test set. SHAP (Shapley Additive Explanations) analysis identified key differential markers, including fructose, turanose, glucose, and GABA, which contributed most to the classification. Furthermore, a user-friendly web application was developed to facilitate rapid on-site authentication. This study provides an innovative technical framework for honey variety discrimination, with potential applications in quality control and anti-fraud practices.

## 1. Introduction

Honey is a nutrient-rich natural food highly regarded for its unique taste and various bioactive properties, including antioxidant and antibacterial activities. Based on the source of nectar collected by bees, honey can be categorized as multifloral or unifloral. Unifloral honey possesses unique plant-derived aromas and specific bioactive components, thus often commanding higher prices in the market. However, not all unifloral honeys share the same characteristics. For instance, rape honey, which is highly similar in color to high-value acacia honey, is often regarded as a representative of low-value unifloral honeys due to its relatively ordinary flavor and less specific components. Unfortunately, fraudulent practices where low-value rape honey is passed off as high-value acacia honey are common in the market and not only jeopardize consumer rights but also disrupt market order [1]. Therefore, developing a precise and efficient method for rape honey and acacia honey identification is crucial for ensuring the integrity of the honey market and enhancing consumer confidence.

At present, honey identification technologies are mainly divided into two major categories: destructive detection and non-destructive detection. Typical representatives of non-destructive identification methods include near-infrared spectroscopy and fluorescence spectroscopy. These methods do not cause damage to the structure or components of honey samples during the detection process and possess significant advantages such as fast analysis speed and convenient operation. However, they are unable to obtain the characteristic components of the samples [2]. Liquid chromatography–tandem mass spectrometry (LC-MS/MS) is a technology widely used to detect sample component information in the field of destructive detection, and it has core advantages such as revealing the species origin of the honey or the specificity during the brewing process. For example, Ali et al. applied metabolomics to reveal the differential characteristics of flavonoids among different honey species [3,4]. Although this method has unique advantages in revealing the overall changes in small-molecule metabolites in samples, its identification process is highly dependent on the matching of reference databases. Nevertheless, the coverage of existing databases is limited, and problems such as uneven spectrum quality and insufficient standard substance information often exist. These issues lead to situations where incorrect matching of non-target substances is prone to occur or isomers are difficult to distinguish in practical analysis, thereby resulting in false-positive identification results [5]. Carbohydrates are the main chemical components of honey, among which glucose and fructose account for approximately 75% of the total carbohydrate content, while the remainder are disaccharides and a small number of other carbohydrates. The carbohydrates in honey determine its properties such as energy value, viscosity, hygroscopicity, and crystallinity. The composition of these carbohydrates mainly depends on the botanical origin of the honey (i.e., the type of flowers collected by bees) and its geographical origin, and it is also affected by factors such as climate, processing methods, and storage conditions [6]. In addition to carbohydrates, amino acids account for 1% of honey components, and their relative proportions depend on the origin of honey, as well as the enzymes derived from microorganisms during the natural fermentation of honey and the secretions from the salivary glands and pharynges of bees [6]. It can be reasonably inferred based on these characteristics that there may be significant differences in the chemical profiles of carbohydrate and amino acid compositions between acacia honey and rape honey [6].

However, relevant studies are scarce. Further research can accurately distinguish high-value acacia honey (i.e., golden acacia honey) from low-value rape honey based on this information. In recent years, machine learning algorithms have been increasingly applied in food quality authentication [7,8] and fraud detection as they can uncover underlying patterns in data from high-dimensional features, thereby achieving accurate sample analysis [9]. Machine learning algorithms are currently widely used in honey variety identification.

For example, Geană, E et al. systematically applied multiple machine learning-related methods such as principal component analysis (PCA) to construct a comprehensive model for honey samples with different adulteration types and concentrations based on ultraviolet–visible (UV-Vis) spectroscopy data. Their method covers both adulteration identification and quantitative analysis of adulteration levels [10]. Mara, A et al. took elemental fingerprints as the characteristic indicators of honey and combined machine learning techniques to solve the challenging problem of identifying the geographical origin of honey [11]. It is well known that the training accuracy of machine learning models is closely related to the acquisition of high-quality datasets. The quality of a dataset is reflected not only in the sufficiency of sample quantity but also in its representativeness, annotation accuracy, feature integrity, and the rationality of data distribution [12,13]. In terms of honey variety identification, the number of samples for each honey variety is only about 10–20 in most studies, which poses a challenge to the accuracy and practicality of honey variety identification models.

Accordingly, a total of 373 acacia honey samples and 169 rape honey samples were collected in this study over two years from major acacia honey and rape honey production areas in China, covering more than 10 provinces, municipalities, and autonomous regions. Secondly, an ultra-high-performance liquid chromatography–tandem mass spectrometry (UPLC-MS/MS) method was established for the quantitative analysis of 12 carbohydrates and 20 amino acids. The differences in carbohydrates and amino acids between acacia honey and rape honey in the large sample dataset were analyzed, and through combination with machine learning techniques, a precise identification method for distinguishing rape honey from acacia honey (i.e., golden acacia honey) was developed.

Machine learning algorithms [8] were used to evaluate their feasibility in distinguishing acacia honey and rape honey, and interpretable machine learning [14] was employed to explore the role of key carbohydrates and amino acids in honey variety identification. This study’s dataset has significant characteristics—A large number of acacia honey and rape honey samples were collected from more than ten provinces, municipalities directly under the Central Government, and autonomous regions across the country, all of which were identified through palynological analysis and expert appraisal. The large sample size and high-quality dataset laid a solid foundation for training an accurate model in this study. Meanwhile, this study innovatively adopted the method of combining sugar and amino acid components with machine learning, which can be used to distinguish honey varieties that cannot be directly distinguished manually, providing a reference for the identification of other high-sugar or high-amino acid food varieties. Overall, we believe these results will provide theoretical guidance for the discrimination and application of rape honey and acacia honey, as well as a reference for the variety identification and anti-fraud practices of other high-value honeys, and are expected to promote the improvement of honey quality control systems.

The main contributions of this paper are summarized as follows:

(i) Integrating the quantification of 12 sugars and 20 amino acids with machine learning, a precise discrimination method for rape and acacia honey was built in this study, addressing traditional limitations and offering a new “chemical fingerprint”-based path for honey authentication.

(ii) Discrimination accuracy and interpretability were enhanced by comparing five machine learning algorithms, optimizing the MLP to achieve 98% and 100% accuracy for the two honeys, and identifying key markers via SHAP.

(iii) A user-friendly web app enabling rapid on-site honey authentication was developed, bridging lab research and practice and supporting honey market quality control and anti-fraud practices.

## 2. Materials and Methods

### 2.1. Chemicals and Reagents

The analytical standards of 20 amino acids, namely, aspartic acid (Asp), glutamic acid (Glu), γ-aminobutyric acid (GABA), serine (Ser), glutamine (Gln), histidine (His), asparagine (Asn), glycine (Gly), threonine (Thr), arginine (Arg), alanine (Ala), tyrosine (Tyr), valine (Val), methionine (Met), tryptophan (Trp), phenylalanine (Phe), isoleucine (Ile), leucine (Leu), lysine (Lys), and proline (Pro), were obtained from Alta Scientific Co. (Tianjin, China), all with purity ≥ 99%. Phenyl isothiocyanate (PITC) (purity ≥ 99%) and triethylamine (purity ≥ 99%) were purchased from Sigma-Aldrich (St. Louis, MO, USA). Hexane (HPLC grade) and acetonitrile (HPLC grade) were obtained from Dikma (Beijing, China). Acetic acid (analytical grade) was obtained from Taicang Hushi Reagent Co. (Ningbo, China).

Fructose, glucose, sucrose, turanose, maltulose, maltose, kojibiose, isomaltose, erlose, melezitose, raffinose, and maltotriose (≥98%) were purchased from Aladdin Scientific Co., Ltd. (Shanghai, China).

### 2.2. Sample Preparation and Derivatization

All honey samples were directly collected from representative beekeeping farms, placed in sealed food-grade containers, and transported to the laboratory under low-temperature conditions within 48 h. Upon arrival, the samples were immediately stored at −20 °C in a dark environment until chemical analysis. This storage protocol was adopted to minimize enzymatic activity and chemical degradation, especially for unstable compounds such as free amino acids and reducing sugars (e.g., glucose and fructose), thereby maintaining sample integrity throughout the study.

The sample pretreatment procedure was performed as described by Yang et al. [15]. Briefly, an aliquot of honey sample (1.0 g) was weighed and transferred into a 50 mL centrifuge tube, and 25 mL of 0.1 mol/L HCl solution was added and then vortexed for mixing. Next, 1 mL of the liquefied honey samples was transferred to a 10 mL centrifuge tube, and 40 μL of the internal standard leucine working solution was added to the tube, followed by the addition of 500 μL of phenyl isothiocyanate and 500 μL of triethylamine. The contents were thoroughly mixed, and the mixture was allowed to stand at room temperature for one hour. Subsequently, 100 μL of aqueous acetic acid was added and mixed well. Then, 2 mL of n-hexane was added, and the mixture was vortex-mixed for 2 min and allowed to stand. After phase separation, the lower layer solution was aspirated and passed through a 0.22 μm microporous filter membrane.

### 2.3. UPLC-MS/MS Analysis Conditions

Amino acid derivatives were analyzed using UPLC-MS/MS equipped with an Atlantis T3 (2.1 mm × 150 mm, 3 μm) column (Waters, Milford, MA, USA) at a column temperature of 25 °C and a flow rate of 0.3 mL/min. The mobile phase consisted of acetonitrile (A) and 10 mmol/L aqueous ammonium acetate (B). The linear gradient program was as follows: 0–1 min, 1% A; 1–2.5 min, 1–20% A; 2.5–8 min, 20% A; 8–9 min, 20–80% A; 9–11 min, 80% A; 11–11.2 min, 80–1% A; and 11.2–14 min, 1% A. The injection volume was 2 μL, and mass spectrometry was carried out in the positive mode with electrospray ionization (ESI). Mass spectrometry (MS) analysis conditions were as follows: capillary voltage of 3.0 kV, lens voltage of 0.1 V, desolvation temperature of 350 °C, source temperature of 120 °C, desolvation gas (N2) flow rate of 650 L/h, cone gas (N2) flow rate of 50 L/h, and collision gas (Ar) flow rate of 0.15 mL/min, Furthermore, the linear range of amino acids generated by the instrument was 1–500 mg/kg.

### 2.4. Determination of Sugar Content

A total of 2.5 g of honey sample (accurate to 0.001 g) was weighed and placed into a 50 mL beaker. Subsequently, 15 mL of water was added, and the mixture was stirred with a glass rod until the sample was completely dissolved. The solution was transferred to a 50 mL volumetric flask, and an additional 20 mL of acetonitrile was added before diluting to the mark with water. The diluted solution was filtered, and the beaker was rinsed with 5 mL of water, which was also transferred to the 50 mL volumetric flask. This rinsing step was repeated three times. The mixture was then mixed thoroughly and passed through a 0.22 μm filter membrane for further analysis. Finally, the filtered solution was placed in a sample bottle for liquid chromatography analysis.

For the analysis of sucrose, turanose, maltulose, maltose, kojibiose, isomaltose, erlose, melezitose, raffinose, and maltotriose, 3 μL of the filtrate was injected into an ultra-performance liquid chromatograph with an evaporative light scattering detector (UPLC-ELSD) system utilizing a BEH Amide column (2.1 × 150 mm, 1.7 μm) (Waters, Dublin, Ireland). Chromatographic separation was performed at a flow rate of 0.25 mL/min at 60 °C. The binary gradient elution system comprised 0.1% triethylamine–acetonitrile (A) and water (B), with separation achieved using the following gradient program: 0 min (10% B), 0~3 min (10% B), 3~10 min (10~20% B), 10~23 min (20% B), 23~26 min (20~35% B), 26~28 min (35% B), 28~29 min (35~10% B), and 29~35 min (10% B). The ELSD was operated in heating nebulizer mode, with a drift tube temperature of 55 °C and gas pressure of 30 psi.

For the analysis of fructose and glucose, 10 μL of the filtrate was injected into a high-performance liquid chromatography device with a refractive index detector (HPLC-RID) system equipped with a BEH Amide column (250 × 4.6 mm, 3.5 μm) (Waters, Ireland). Chromatographic separation was performed at a flow rate of 0.1 mL/min, and the column temperature was maintained at 35 °C. The mobile phase was 0.1% triethylamine in acetonitrile/water (75:25, *v*/*v*).

Standard solutions of fructose and glucose with concentrations of 0.8 g/100 g, 1.2 g/100 g, 1.6 g/100 g, 2.0 g/100 g, and 2.4 g/100 g as well as mixed standard solutions containing 10 oligosaccharides with concentrations of 0.01 g/100 g, 0.02 g/100 g, 0.05 g/100 g, 0.1 g/100 g, and 0.15 g/100 g were prepared using 40% acetonitrile–water. Specifically, the linear range for fructose and glucose was 16–48 g/100 g, while the linear range for other ten oligosaccharides was 0.2–3.0 g/100 g, which matched the concentration gradients of the prepared standard solutions. The linear equations and correlation coefficients for the 12 sugar components are listed in Table 1.

### 2.5. Machine Learning Modeling and Analysis

#### 2.5.1. Data Preprocessing

A total of 32 features were constructed by concatenating the concentrations of 12 sugars and 20 amino acids, yielding the input variable matrix X = [x_1_, x_2_, x_3_, …, x_32_]. Prior to modeling, each feature in X was standardized using the transformation function x′ = (x − μ)/σ, where μ denotes the mean and σ the standard deviation across all samples. The resulting normalized matrix is denoted as X′. For subsequent classification tasks, rape honey and Acacia honey samples were assigned binary class labels of 0 and 1, respectively.

#### 2.5.2. Unsupervised Dimensionality Reduction and Visualization

Principal component analysis (PCA), t-distributed stochastic neighbor embedding (t-SNE), and uniform manifold approximation and projection (UMAP) are commonly used dimensionality reduction algorithms [16]. By projecting high-dimensional data into lower-dimensional spaces, these techniques facilitate the interpretation of relationships among data features of different sample types and are widely employed for dataset feature visualization [17]. In this study, PCA, t-SNE, and UMAP were applied to the standardized feature matrix X′, comprising sugar and amino acid profiles, to generate two-dimensional visualizations for rape honey and Acacia honey samples.

#### 2.5.3. Classification Models

Five machine learning algorithms (Decision Tree, Gaussian Naïve Bayes (GaussianNB), Linear Discriminant Analysis (LDA), Light Gradient Boosting Machine (LightGBM), k-Nearest Neighbors (KNN), and Multilayer Perceptron (MLP)) were employed to construct binary classifiers [18,19] for distinguishing rape honey from Acacia honey. These algorithms have been widely used in prior studies for the classification and geographical authentication of agricultural products and honey varieties [19].

The dataset was partitioned into a training set and a validation set in an 80%:20% ratio [4,20,21]. Specifically, stratified sampling was conducted to retain the original class proportion, and the test set was isolated throughout the process to prevent data leakage. By comparing the content distribution of various sugars and amino acids in rapeseed honey and acacia honey in the training set and test set, it was proved that the overall distribution of the data set division was highly consistent, and no information leakage occurred (Figures S1 and S2). Model training was performed using 10-fold cross-validation on the training set, and each fold was used as the validation set in turn. The mean and low standard deviation of the indicators were calculated. Meanwhile, the Adaptive Synthetic Sampling (ADASYN) technique was only applied to the training data of each fold to address class imbalance and enhance model performance, while the validation set and the test set maintained the original distribution [22].

In addition, hyperparameter tuning was performed for each model to improve model stability through RandomizedSearchCV (Table 2). Considering the moderate input feature dimension in this study, and through cross-validation, it was finally determined that a single hidden layer with 8 neurons can maintain optimal fitting. Meanwhile, various commonly used activation functions were compared and tested, and it was found that the sigmoid function has strong interpretability of output probability in binary classification tasks and stable convergence on small-sample datasets. Regarding the selection of the optimizer, the L-BFGS algorithm performs iterative optimization through the approximate Hessian matrix, which can not only ensure convergence accuracy but also reduce the number of iterations in the scenario of this study. In addition, we introduced L2 regularization to avoid overfitting, selected 0.01 as the regularization coefficient, and set the maximum number of iterations to 300.

We adapted the architecture of the Multilayer Perceptron (MLP) (input → hidden → output layers), a feedforward neural network, to our binary classification (k = 2) of honey. For input honey feature vector x, hidden layer computation (with sigmoid activation $\sigma (z)=\frac{1}{1+e^{-z}}$) is $h=\sigma (W_{1}x+b_{1})$, and output layer computation is $\hat{y}=\sigma (W_{2}h+b_{2})$, where $\hat{y}$ gives class probabilities. Cross-entropy loss, which guides optimization, is defined as

$$L=-\frac{1}{N}\sum_{i=1}^{N}\sum_{j=1}^{2}y_{ij}\ln({\hat{y}}_{ij})$$

(N: sample count; $y_{ij}$: true label; ${\hat{y}}_{ij}$: predicted probability). We used the L-BFGS algorithm [23] for efficiency on small datasets.

A feedforward neural network with two hidden layers [23] was implemented in the MLP model. The first hidden layer contained five neurons, and the second contained two neurons. Logistic (sigmoid) functions were used as activation functions in both hidden layers. The network was optimized using the limited-memory Broyden–Fletcher–Goldfarb–Shanno (L-BFGS) algorithm [24], which is particularly suited for small-sample datasets. After training, each model’s predictive performance was evaluated using the 20% holdout test set. Only the training set was subjected to ADASYN oversampling in this study, and the test set remained independent and unaffected. L2 regularization was introduced into the MLP model to suppress overfitting that may be caused by oversampling; 10-fold cross-validation was adopted instead of a single validation set to reduce the impact of sampling bias on the results through multiple random divisions, making the performance evaluation results more statistically reliable, thereby avoiding potential risks during oversampling and effectively suppressing overfitting.

#### 2.5.4. Evaluation Criteria

To evaluate the predictive performance of each classification model, confusion matrices were generated for both the training and validation sets, yielding the counts of true positives (TPs), false positives (FPs), false negatives (FNs), and true negatives (TNs). Based on these values, five standard performance metrics were calculated according to Equations (1)–(5): accuracy (ACC), sensitivity (Sn), specificity (Sp), precision, and the Matthews correlation coefficient (MCC) [16].

The metrics ACC, Sn, Sp, and precision range from 0 to 1. A higher ACC indicates better overall classification performance; Sn reflects the model’s ability to correctly identify positive instances, with values closer to 1 indicating stronger performance; Sp measures the ability to correctly identify negative instances; and higher precision indicates greater reliability in positive class predictions. The MCC, ranging from −1 to 1, provides a balanced assessment even for imbalanced datasets: a value of 1 indicates perfect prediction, 0 represents random classification, and −1 denotes complete disagreement between prediction and observation. Models with MCC values closer to 1 are considered to exhibit superior predictive performance.

(1) $$\mathrm{ACC}=\frac{\mathrm{TP}+\mathrm{TN}}{\mathrm{TP}+\mathrm{FP}+\mathrm{FN}+\mathrm{TN}}$$

(2) $$\mathrm{Sn}=\frac{\mathrm{TP}}{\mathrm{TP}+\mathrm{FN}}$$

(3) $$\mathrm{Sp}=\frac{\mathrm{TN}}{\mathrm{TN}+\mathrm{FP}}$$

(4) $$\mathrm{Precision}=\frac{\mathrm{TP}}{\mathrm{TP}+\mathrm{FP}}$$

(5) $$\mathrm{MCC}=\frac{\mathrm{TP}\;\times \;\mathrm{TN}\;-\;\mathrm{FP}\;\times \;\mathrm{FN}}{\sqrt{(\mathrm{TP}+\mathrm{FP})(\mathrm{TP}+\mathrm{FN})(\mathrm{TN}+\mathrm{FP})(\mathrm{TN}+\mathrm{FN})}}$$

#### 2.5.5. Interpretable Machine Learning

To improve the interpretability of the optimal machine learning model, we adopted SHAP (Shapley Additive Explanations) to provide both global and local explanations [25] of model behavior [26]. SHAP is a unified framework based on Shapley values from cooperative game theory [27], capable of quantifying the marginal contribution of each feature to the model’s output. In this study, the SHAP Kernel Explainer module was employed, using a subset of the training data as background to compute SHAP values for the test samples. These values represent the contribution of each feature to the model’s prediction at the individual level. A summary plot was generated to visualize global feature importance across the dataset [28], while local explanations were examined to elucidate model decisions on a per-sample basis. This approach enhances the transparency of the model and facilitates interpretation of its predictive behavior [29].

### 2.6. Statistical Analysis

Statistical significance analyses were conducted using the SPSS software package (version 17.0). Experimental data are expressed as mean ± SD (mean plus and minus standard deviation). Differences in compound concentrations between groups were assessed using Student’s *t*-test implemented in the scipy library, with a *p*-value < 0.05 considered statistically significant. Hierarchical clustering heatmaps were generated using the matplotlib and seaborn libraries. All machine learning procedures and analyses were performed in Python 3.7 using the Scikit-Learn library [30].

## 3. Results and Discussion

### 3.1. Sample Information and Source Description

**Figure 1:**
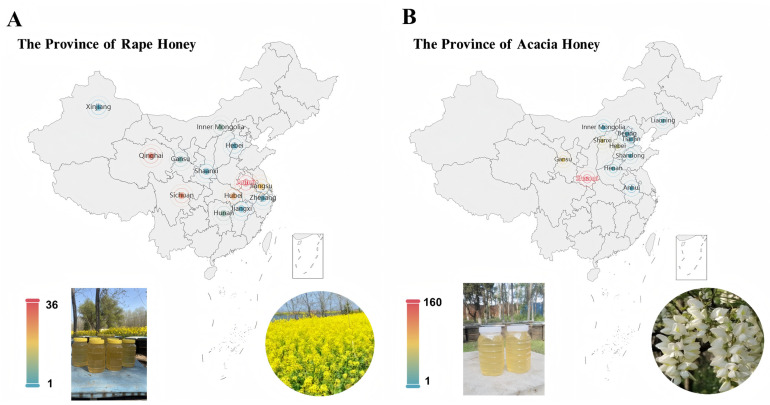
Information on samples of rape honey and acacia honey originating from different provinces of China. (**A**) Rape honey; (**B**) Acacia honey.

A representative honey sample dataset serves as a fundamental prerequisite for investigating the differences between high-value acacia honey and low-value rape honey. To this end, we collected a large number of acacia honey and rape honey samples from major beekeeping farms across China. Specifically, 373 acacia honey samples were collected from 11 provincial-level administrative regions, namely, Shaanxi, Gansu, Shanxi, Hebei, Liaoning, Henan, Shandong, Beijing, Inner Mongolia, Anhui, and Tianjin. In addition, 169 rape honey samples were collected from 13 provinces and autonomous regions, namely, Anhui, Qinghai, Sichuan, Hubei, Jiangsu, Inner Mongolia, Hunan, Gansu, Zhejiang, Shaanxi, Hebei, Jiangxi, and Xinjiang. These samples were collected from representative beekeeping farms located in major monofloral nectar source areas, and all honey varieties were identified through palynological analysis and expert appraisal. The source information of these samples is shown in Figure 1. For honey from different regions, we conducted statistical analysis on amino acid composition and sugar contents (such as fructose and glucose). As shown in Supplementary Materials File S1, analysis of variance (ANOVA) revealed differences in different indicators of the same type of honey among regions. It can be seen from the table that the honey components involved in this study have obvious geographical characteristics, which also explains the objective reason for the inhomogeneity of sample distribution from the perspective of chemical composition, further confirming data heterogeneity.

### 3.2. Amino Acid and Sugar Composition Analysis

The main components of honey are carbohydrates—fructose, glucose, sucrose, and other sugars. The proportion of these sugars among nectars varies among different plants. Amino acids originate from trace proteins and free amino acids inherent in plant nectar, as well as enzymatic substances secreted by bees during collection and brewing, exhibiting species specificity. Therefore, the types and contents of sugars and amino acids constitute the “chemical fingerprint” for distinguishing honey varieties and verifying authenticity. Previous studies have all demonstrated that there are differences in amino acid contents among different honey varieties, and amino acid profiles can be used as variables to construct models for the identification of honey origin [6].

**Figure 2:**
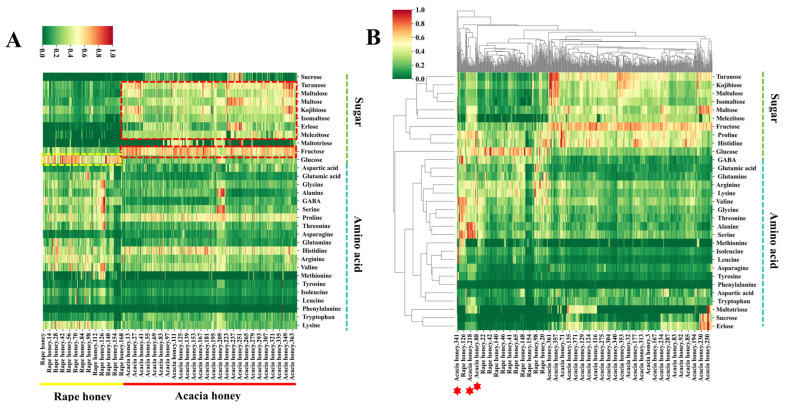
Heatmap and clustered heatmap of sugars and amino acids in rape honey and acacia honey. (**A**) Heatmap; (**B**) clustered heatmap.

**Figure 3:**
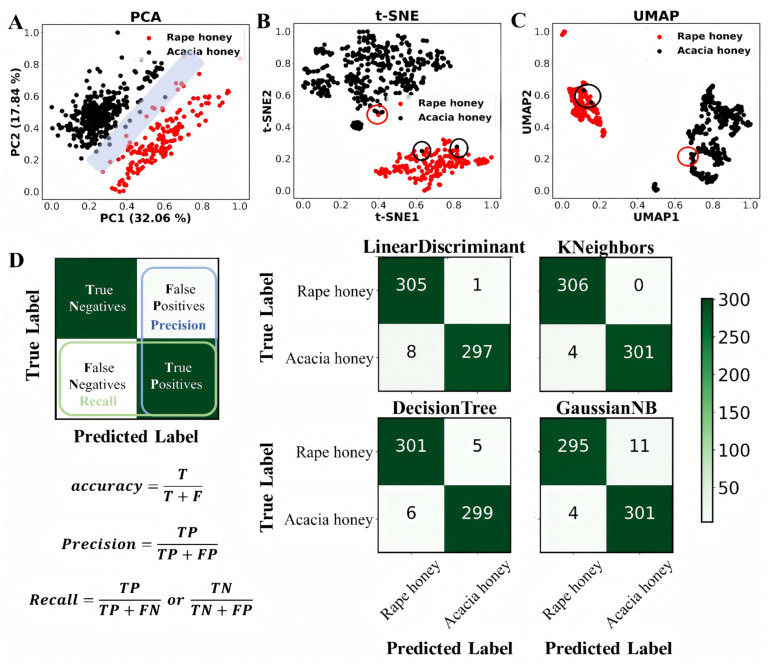
Distinguishing between rape honey and acacia honey based on unsupervised and supervised learning. (**A**–**C**) Plots of PCA, t-SNE, and UMAP analyses based on normalized feature data, respectively, and (**D**) results of confusion matrices of four supervised learning classification algorithms in conjunction with the ADASYN oversampling method for 10-fold cross-validation in 80% of the dataset [31].

To investigate the differences in sugars and amino acids between acacia honey and rape honey, we employed UPLC-MS/MS to determine the contents of 12 monosaccharides and oligosaccharides as well as 20 amino acids in 542 honey samples, followed by differential analysis. The results are presented in Figure 2. Figure 3A shows a heatmap of sugar and amino acid contents in acacia honey and rape honey, from which significant differences in the contents of certain sugars and amino acids between the two types of honey can be clearly observed.

In terms of sugar contents, turanose, maltulose, maltose, kojibiose, isomaltose, erlose, melezitose, and fructose were found at higher levels in the majority of acacia honey samples, whereas glucose content in rape honey samples was higher than in most acacia honey samples. Except for raffinose, all sugars exhibited significant differences between the two types of honey (*p*-value < 0.05) (Figure S3). These differences in sugar contents are related to variations in the synthesis, metabolism, and accumulation of sugars in nectars among different plants, among other factors.

The mechanisms of sugar synthesis (mediated by SPS) and transport (mediated by SWEET9) in plants directly determine the type and content of sugars in nectar, which may further affect the subsequent differences in sugar composition of honey. For example, sucrose in nectar is catalytically synthesized by sucrose phosphate synthase (SPS) in the parenchyma cells of nectaries and then secreted into the extracellular space through the sugar transporter SWEET9. Subsequently, it is hydrolyzed into glucose and fructose by extracellular invertase, ultimately forming nectar with different proportions of sugars.

This difference in nectar sugar composition is not only related to the structural location of nectaries and the characteristics of nectar secretion; there are significant differences in the type and location of nectaries among different plants, and these differences are associated with the sugar composition of nectar. Nectaries at different locations exhibit distinctions in sugar secretion patterns. The developmental maturity of nectaries also affects the characteristics of nectar secretion: as nectaries mature, the expression level of SWEET9 increases, reaching the highest level during the peak period of nectar secretion. Moreover, the dynamics of starch accumulation and degradation in the parenchyma cells of nectaries (starch accumulation before flowering and degradation into sugars for secretion after flowering) further regulate the sugar release amount of nectar. These differences in nectary structural location and nectar secretion characteristics lead to variations in sugar content and proportion in the nectar of different plants, which may ultimately result in differences in the sugar content of honey [32,33]. For instance, the nectary structure of rape is relatively simple, and its secretion mechanism relies on the hydrolysis of sucrose transported via sieve tubes into monosaccharides [10,25,34].

Regarding amino acid contents, all amino acids except for tyrosine and phenylalanine showed significant differences between the two types of honey (*p*-value < 0.05) (Figure S4). The heatmap results indicated that the contents of glycine, GABA, threonine, and valine in some rape honey samples were much higher than the overall levels in acacia honey, and such differences may be associated with local ecological environments [35]. Notably, most acacia honey and rape honey samples can be distinguished based solely on sugar and amino acid contents, but a small portion of samples from the two types exhibit high similarity, leading to incomplete identification (Figure 2B) [36].

### 3.3. Machine Learning Analysis

Three dimensionality reduction methods, namely, PCA, t-SNE, and UMAP, were employed for visualization analysis of rape honey and acacia honey, with the results shown in Figure 3A–C. It can be clearly observed that all three methods could distinguish most rape honey and acacia honey samples, indicating that sugars and amino acids can be used for subsequent supervised learning classification modeling. Among the three dimensionality reduction methods, two (t-SNE and UMAP) achieved better differentiation performance than PCA. This is because both belong to the category of manifold learning, which can reveal the underlying non-linear structure of data and present clear cluster structures and local density distributions of the data.

**Figure 4:**
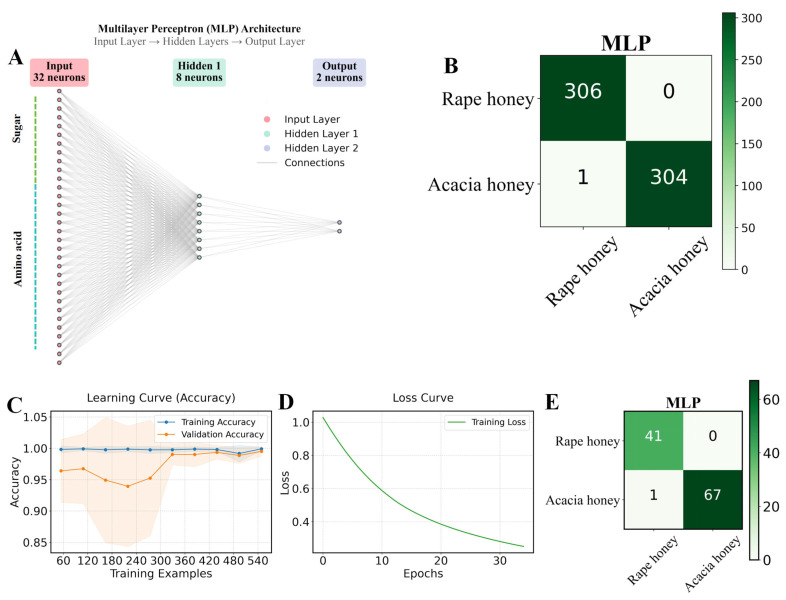
Distinguishing between rape honey and acacia honey based on the MLP algorithm. (**A**) diagram of the MLP architecture; (**B**) confusion matrix results of the MLP algorithm combined with the ADASYN oversampling method for 10-fold cross-validation in 80% of the dataset; (**C**,**D**) representation of the MLP algorithm’s learning and loss curves, respectively; and (**E**) confusion matrix results of the optimal MLP model for 20% of the test set.

Furthermore, five supervised classification machine learning algorithms combined with the ADASYN oversampling method were used for 10-fold cross-validation training on 80% of the dataset, and the results are presented in Figure 3D and Table 3. ACC (accuracy) describes the consistency between the model’s predicted results and the true labels in the statistical dataset. A higher ACC value indicates higher model accuracy and better performance of the classification model. Among the five algorithms, the MLP (Multilayer Perceptron) algorithm achieved the highest ACC of 0.998, outperforming the other four models. In addition, the Sn (sensitivity), Sp (specificity), MCC (Matthews correlation coefficient), AUC (Area Under the Curve), precision, and F1-score of the MLP algorithm were 0.996, 1, 0.997, 1, 1, and 0.999, respectively. Among these, the Sn, MCC, and F1-score were superior to those of the other models, while the Sp, AUC, and precision were the same as those of the KNeighbors algorithm (Table 3). In the confusion matrix, the MLP model outperformed the other models in recognition and classification, achieving 100% recognition of rape honey with a small error rate in acacia honey recognition (Figure 4A,B).

**Figure 5:**
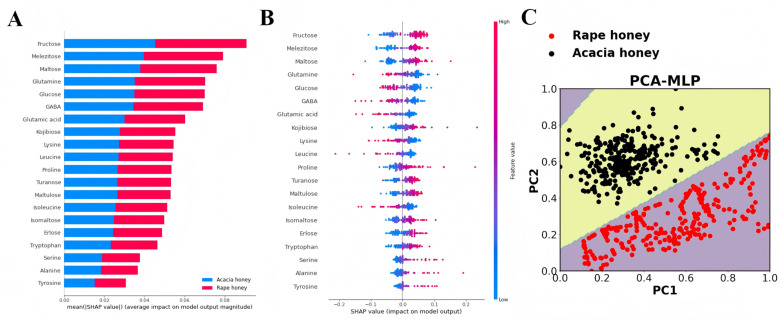
Model interpretability analysis based on MLP algorithm combined with SHAP. (**A**) Summary plot of rape and acacia honey; (**B**) output of feature importance of acacia honey based on SHAP method; and (**C**) visualization of MLP decision boundary based on PCA.

**Figure 6:**
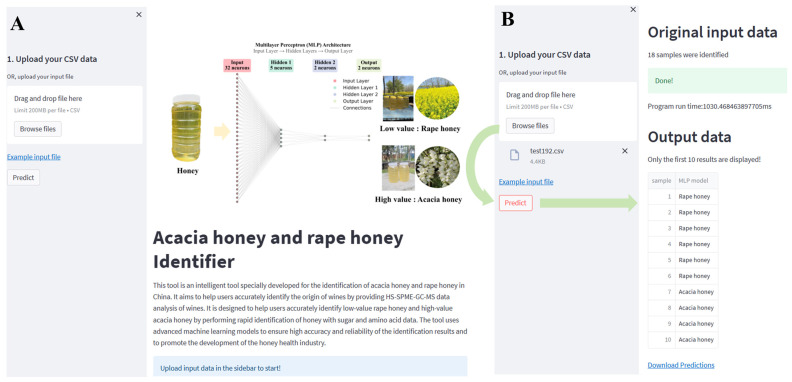
MLP-based web app for recognizing acacia honey and rape honey. (**A**) Web app schematic; (**B**) usage process.

As shown in Figure 4C,D, the training loss of the MLP algorithm continuously decreased and stabilized, while the learning curves of the training set and validation set gradually approached and stabilized, indicating that the model was effectively learning with good generalization performance. The high accuracy of the MLP in distinguishing rape honey and acacia honey compared with other algorithms is closely related to its strong non-linear representation ability and automatic feature learning ability [9]. In the 20% test set, the MLP model achieved the highest AUC value (0.997) among the five algorithms, while the remaining indicators were consistent with those of the Linear Discriminant and GaussianNB models (Table 4). According to the confusion matrix of the test set, the MLP achieved 100% recognition of rape honey, with only 1 sample misidentified among 68 acacia honey samples. As can be seen from Figure 5 and Figure 6, the two types of honey have significant differences in sugar and amino acid compositions, while the test set and training set have similarities; although there are slight fluctuations in the contents of the same type of honey between the training set and the test set, the overall distribution is highly consistent, indicating that the data division is reasonable and no information leakage has occurred. It should be clarified that other models (such as Linear Discriminant, KNN, Decision Tree, and GaussianNB) did not perform poorly, but achieved similar performance on the training set and test set, which was only slightly lower than that of the Multilayer Perceptron (MLP). This may be because the sugar and amino acid contents of honey are continuous and high-precision stoichiometric data, which are suitable for models like MLP that can model smooth nonlinear relationships; while decision tree-based methods rely on feature segmentation, which may be slightly rough in capturing subtle concentration gradients. In summary, the MLP classification model trained based on the chemical profiles of sugars and amino acids in this study can accurately identify rape honey and acacia honey.

### 3.4. Interpretability Analysis

The SHAP method was employed for interpretability analysis of the MLP model, with the results shown in Figure 5A,B. Figure 5A presents the mean absolute values of SHAP values for each feature in rape honey and acacia honey, where a larger value indicates greater importance of the feature. Among the top 13 important compounds (mean|SHAP value| > 0.04), carbohydrates account for 61.53%, and the top 6 compounds are all carbohydrates, namely, fructose, turanose, glucose, melezitose, maltulose, and erlose.

A further analysis of the relationship between feature value magnitude and predictive impact is presented in Figure 5B. Each point represents a sample; red points indicate a larger feature value, while blue points indicate a smaller feature value. The results show that the red points for fructose, turanose, melezitose, maltulose, and erlose are mainly concentrated in the region where SHAP values are greater than 0, indicating that higher contents of these compounds are generally associated with acacia honey. In contrast, higher contents of glucose, lysine, and GABA (with SHAP values < 0) are identified by the model as rape honey. These results are consistent with the significance analysis results of sugar and amino acid contents (Figures S1 and S2).

Furthermore, the visualization results of the PCA-MLP decision boundary demonstrate that the MLP model can accurately identify rape honey and acacia honey.

### 3.5. Web App Development

We developed a web app (https://github.com/panernie/HoneyID/, accessed on 22 December 2025) with a web-based interface using the Streamlit framework (https://streamlit.io/) (Figure 6A) in order to facilitate practical applications to quickly differentiate acacia honey from rape honey and control honey fraud. The interface allows the user to upload a CSV file of sugar and amino acid data of the honey. After submitting the file, the classification task can be initiated by clicking on the “Predict” button, as shown in Figure 6B, and the classification results will be visualized in an interactive dashboard. Detailed usage operations are shown in Supplementary Video S1.

## 4. Conclusions

A precise method for discrimination between rape honey and acacia honey was established in this study by targeting 12 sugars and 20 amino acids in 542 samples collected from more than ten provinces, municipalities directly under the Central Government, and autonomous regions across the country, combined with machine learning techniques. Multivariate analysis revealed significant differences in chemical profiles between the two honey types, with fructose, turanose, glucose, and γ-aminobutyric acid (GABA) identified as key discriminatory markers. Among six machine learning algorithms, the optimized Multilayer Perceptron (MLP) model achieved the best performance, with 98% and 100% accuracy for rape honey and acacia honey on the independent test set, respectively. SHAP interpretability analysis further validated the reliability of model decisions. Additionally, a web application was developed using the Streamlit framework and enabled rapid on-site identification, providing a practical tool for quality control in real-world scenarios.

This study is an attempt to integrate the “chemical fingerprints” of sugars and amino acids with the MLP for honey variety discrimination and overcome traditional methods’ limitations, such as subjectivity and inefficiency. The interpretability analysis clarified the role of key markers, offering a scientific basis for honey authenticity verification. It is proved that the combination of sugar and amino acid components with machine learning can be used to distinguish honey varieties that cannot be directly distinguished manually, which provides a reference for the identification of other high-sugar or high-amino acid food varieties, with significant practical implications for regulating the honey market and combating fraud. It also provides a reference for technological innovation in food traceability and quality control.

It is worth noting that the model developed in this study is specifically designed to distinguish between rape honey and acacia honey. As the dataset only includes the sugar and amino acid characteristics of these two types of honey, it is not applicable to the identification of other honey varieties. In the future, we will expand our research on this basis and build a precise identification model covering more honey categories.

## Figures and Tables

**Table 1:** Linear regression equations and correlation coefficients of 12 sugar components.

Sugar Component	Linear Regression Equation	Correlation Coefficient (R^2^)
Fructose	y = 1.29x + 2.40	0.9875
Glucose	y = 1.33x + 3.18	0.9839
Sucrose	y = 1.53x + 7.59	0.9906
Turanose	y = 1.53x + 7.61	0.9904
Maltulose	y = 1.69x + 7.69	0.9882
Maltose	y = 1.73x + 7.72	0.9896
Kojibiose	y = 1.57x + 7.57	0.9877
Isomaltose	y = 2.00x + 7.83	0.9922
Erlose	y = 1.72x + 7.51	0.9890
Melezitose	y = 1.84x + 7.83	0.9927
Raffinose	y = 1.63x + 7.48	0.9827
Maltotriose	y = 1.69x + 7.51	0.9891

**Table 2:** Optimal hyperparameters for classifiers.

Algorithm	Optimal Parameters
MLP	activation = sigmoid, Hidden_layer_sizes = (8, 1), solver = lbfgs
GaussianNB	var smoothing = 0
KNN	leaf size = 48, metric = manhattan, n neighbors = 6, p = 1, weights = distance
Decision Tree	ccp alpha = 0.09, class weight = None, criterion = entropy, max depth = 14, max features = log2, min samples leaf = 1, min samples split = 17, splitter = best
LDA	shrinking = True solver = eigen, shrinkage = 0.74, priors = None, alpha = 0.00

**Table 3:** The performance of five supervised learning classification algorithms on an 80% training set.

80% Train Set 10cv	MLP	Linear Discriminant	KNeighbors	Decision Tree	GaussianNB
ACC	0.998 ± 0.005	0.985 ± 0.016	0.993 ± 0.008	0.982 ± 0.014	0.975 ± 0.021
Sn	0.996 ± 0.010	0.976 ± 0.031	0.987 ± 0.016	0.981 ± 0.022	0.987 ± 0.017
Sp	1 ± 0	0.997 ± 0.01	1 ± 0	0.984 ± 0.017	0.966 ± 0.036
MCC	0.997 ± 0.010	0.971 ± 0.031	0.987 ± 0.017	0.964 ± 0.028	0.952 ± 0.04
AUC	1 ± 0	0.997 ± 0.01	1 ± 0	0.982 ± 0.014	0.996 ± 0.01
Precision	1 ± 0	0.997 ± 0.011	1 ± 0	0.984 ± 0.017	0.964 ± 0.039
f1	0.999 ± 0.003	0.991 ± 0.01	0.997 ± 0.004	0.983 ± 0.014	0.97 ± 0.029

**Table 4:** The performance of five supervised learning classification algorithms on a 20% test set.

20% Test Set	MLP	Linear Discriminant	KNeighbors	Decision Tree	GaussianNB
ACC	0.991	0.991	0.982	0.945	0.991
Sn	0.976	0.976	0.953	0.907	0.976
Sp	1	1	1	0.97	1
MCC	0.981	0.981	0.962	0.884	0.981
AUC	0.997	0.990	0.993	0.946	0.991
Precision	1	1	1	0.951	1
f1	0.996	0.995	0.991	0.948	0.995

## Data Availability

The original contributions presented in the study are included in the article/Supplementary Materials, further inquiries can be directed to the corresponding authors.

## Supplementary Materials

The following supporting information can be downloaded at https://www.mdpi.com/article/10.3390/foods15010070/s1, Figure S1. Differences in the contents of different sugars in rape honey and acacia honey between the training set (A) and the test set (B); Figure S2. Differences in the contents of different amino acids in rape honey and acacia honey between the training set (A) and the test set (B); Figure S3. Differences in the contents of different sugars in rape honey and acacia honey; Figure S4. Differences in the contents of different amino acids in rape honey and acacia honey; File S1: Differences in sugar and amino acid components of acacia honey and rape honey from different producing areas.
