# Supplementary figures and images for: Precise Discrimination Between Rape Honey and Acacia Honey Based on Sugar and Amino Acid Profiles Combined with Machine Learning
Source: Foods. 2025 Dec 25;15(1):70. doi: 10.3390/foods15010070 (PMC12785547; doi:10.3390/foods15010070)

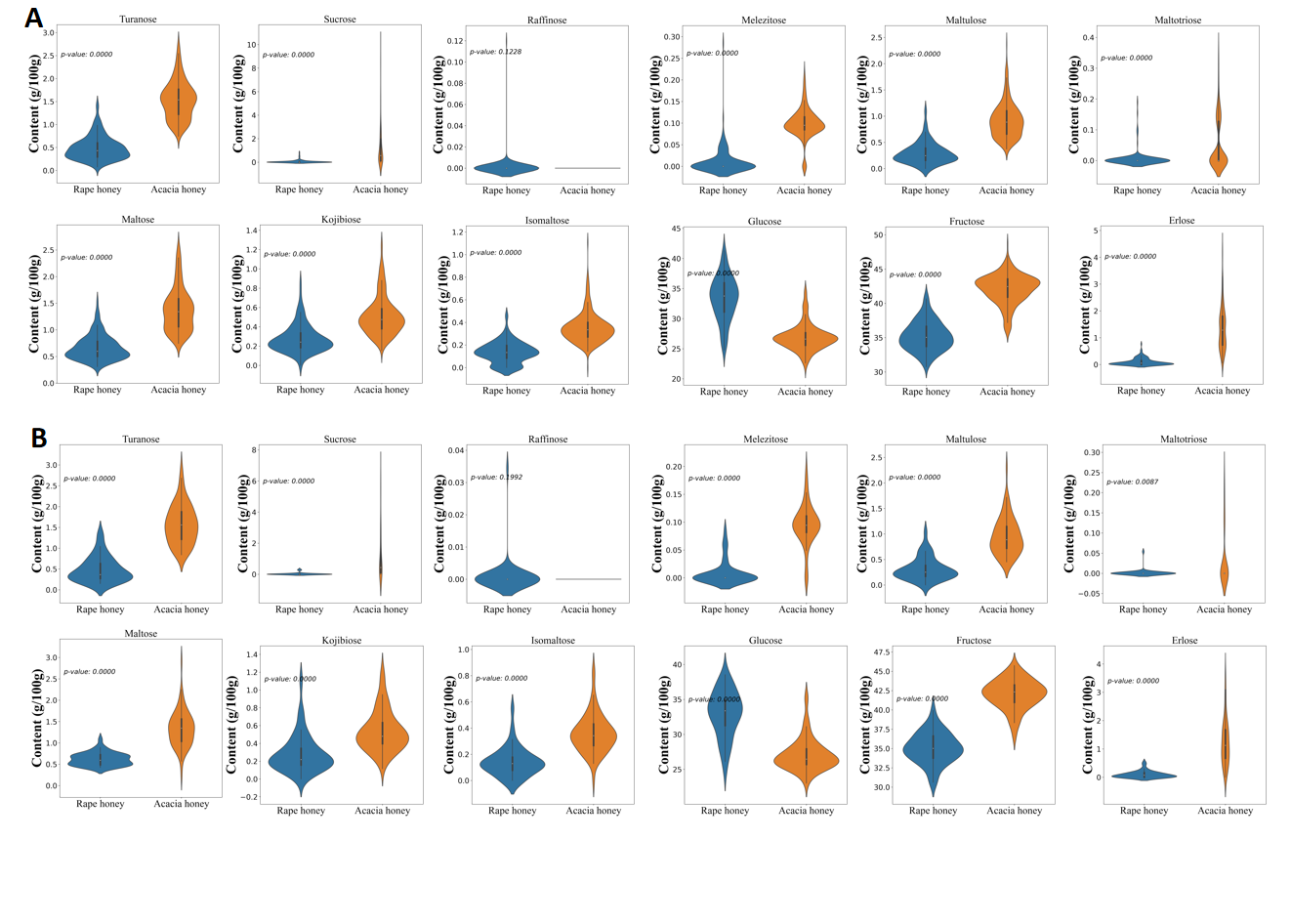

Supplement: Supplementary file 1 [file foods-15-00070-s001.zip › Figure S1.tif]

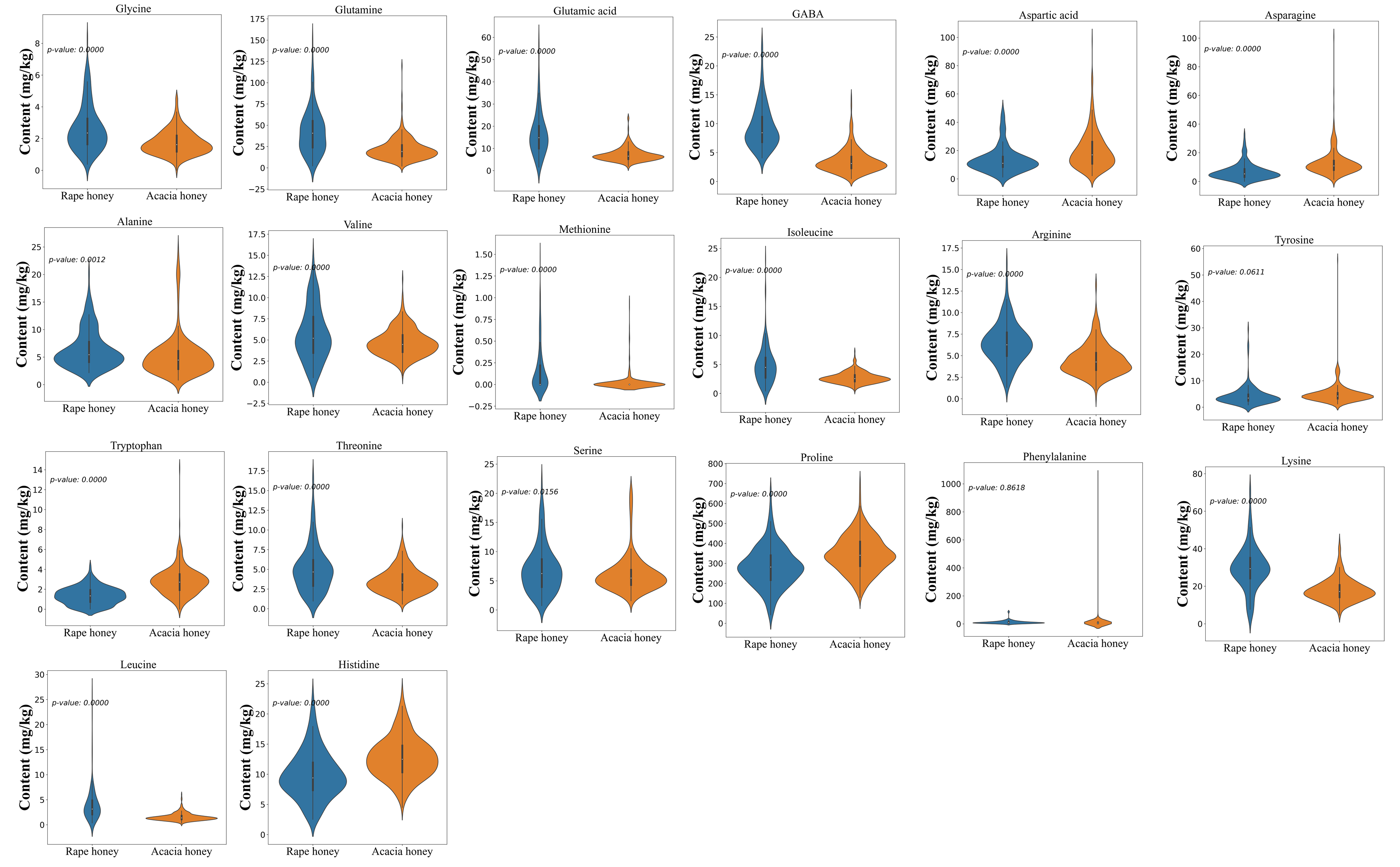

Supplement: Supplementary file 1 [file foods-15-00070-s001.zip › Figure S3.png]

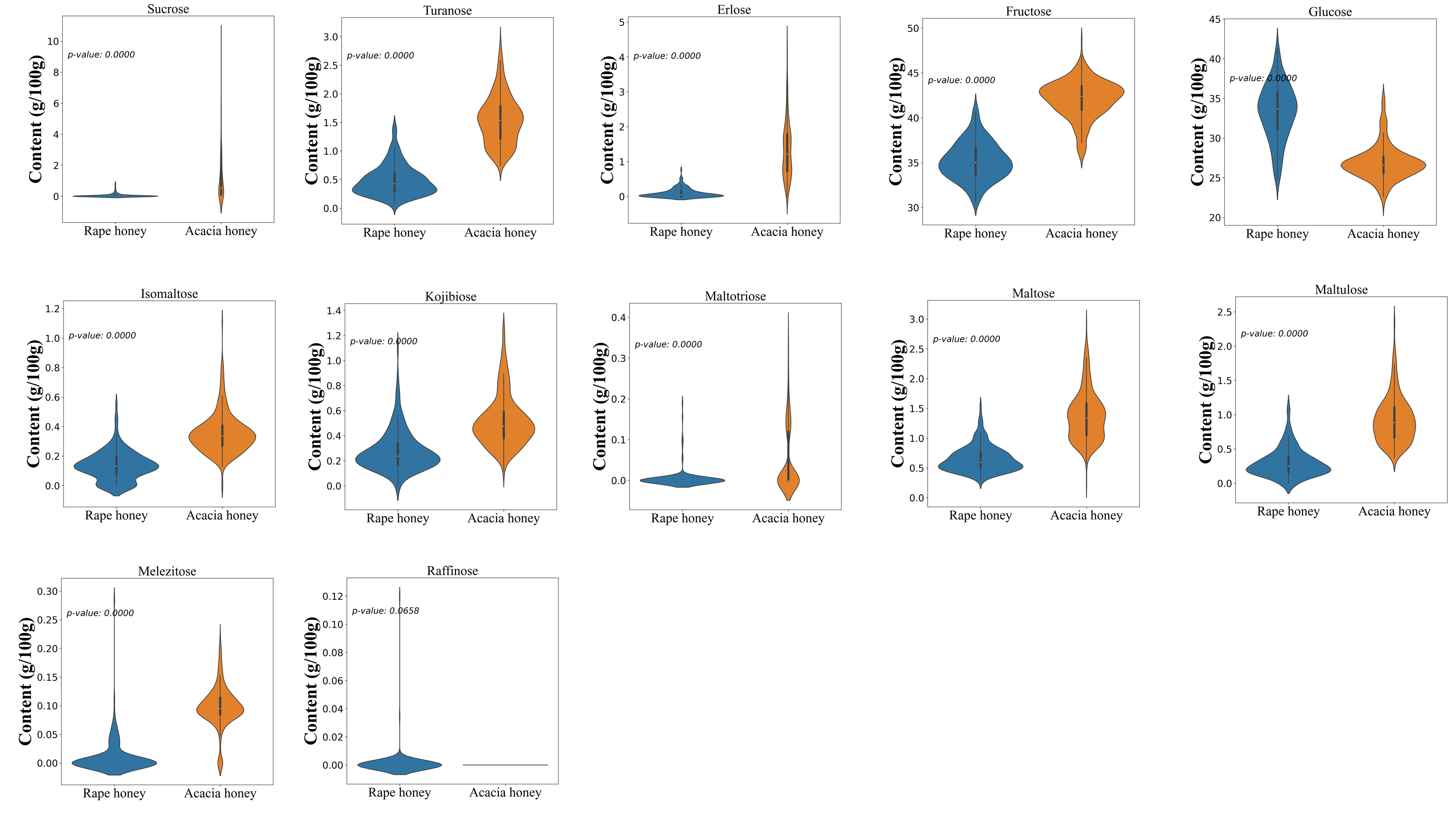

Supplement: Supplementary file 1 [file foods-15-00070-s001.zip › Figure S4.png]
